# A Case of Myelodysplastic Syndrome in an Adult with Down Syndrome: A Rare Observation of a Well-known Pediatric Disease

**DOI:** 10.4274/tjh.galenos.2020.2019.0397

**Published:** 2020-05-06

**Authors:** Harpreet Virk, Shano Naseem

**Affiliations:** 1 Postgraduate Institute of Medical Education and Research, Senior Resident, Department of Pathology, Chandigarh, India; 2 Postgraduate Institute of Medical Education and Research, Department of Hematology, Chandigarh, India

**Keywords:** Adult, Down syndrome, Myelodysplastic syndrome

## To the Editor,

**Figure 1:**
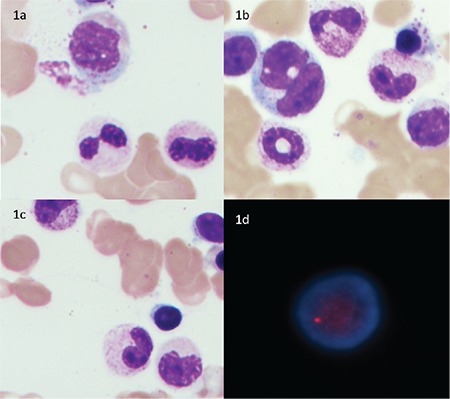
Bone marrow examination revealed significant dysgranulopoiesis in 43% of neutrophils in the form of hypolobation, hypogranulation, ring forms, and pseudo-Pelger-Huet anomaly (a-c), while fluorescence in situ hybridization testing revealed deletion of the 20q12 locus in 140/200 (70%) of nuclei examined (d).

A 56-year-old man with Down syndrome (DS) presented with a fever for the previous 7 weeks. On evaluation he was found to have bicytopenia with hemoglobin (Hb) of 87 g/L and platelet count of 100x10^9^/L. Total leukocyte count was 5.9x10^9^/L. Even after an adequate trial of hematinics, the anemia persisted. Bone marrow examination revealed significant dysgranulopoiesis in 43% of neutrophils in the form of hypolobation, hypogranulation, ring forms, and pseudo-Pelger-Huet anomaly (Figures 1a-1c). No significant dyserythropoiesis or dysmegakaryopoiesis was noted. Bone marrow biopsy showed hypercellular marrow spaces with granulocytic hyperplasia; however, megakaryocytes and erythroid series were adequately represented. Fluorescence in situ hybridization (FISH) testing was performed, which revealed deletion of the 20q12 locus in 140/200 (70%) of nuclei examined (Figure 1d). It was negative for -7/7q deletion, -5/5q deletion, and trisomy 8. The patient currently remains under observation with hematinic supplementation and close follow-up of blood counts.

The age of onset for myeloid neoplasms in children with DS is bimodal, peaking first in the newborn period and again at 3-6 years. However, this increased risk continues even into adulthood [1]. Pertaining to this increased risk, even minor but persistent cytopenias give rise to concerns regarding the possibility of underlying myelodysplastic syndrome (MDS) or marrow failure, or the potential for the development of myeloid leukemia. This has become important given the increasing life expectancy of adults with this disorder [2]. Although conditions like hypothyroidism, obesity, epilepsy, dementia, and Alzheimer’s disease are known to become increasingly prevalent in individuals with DS in later life[3], myeloid leukemias in general and MDS in particular are relatively less common.

Deletion of the long arm of chromosome 20 (del20q) has been reported in 3%-7% of patients with MDS. Isolated del20q is associated with a low risk of progression to AML, with good prognosis and overall prolonged survival [4]. Although this abnormality has been well documented in the MDS subpopulation, its prevalence in MDS associated with DS has not been described in the literature. McLean et al. studied 9 patients with DS with a median age of 41 years having clinical suspicion of MDS. In their cohort, multilineage dysplasia was observed in one case only. No acquired cytogenetic abnormality was seen in any of the cases [5].

Our patient had bicytopenia to begin with, with improvement in leukocyte and platelet counts after supportive care. However, his Hb was persistently low even after an adequate trial of hematinics. Morphological dysplasia was noted in a single lineage in the form of dysgranulopoiesis and FISH studies revealed deletion in 20q, which to our knowledge has never been reported before in this clinical scenario.

DS can no longer be considered a “pediatric” disease; rather, it is a condition that can affect an individual’s whole lifespan. Comprehensive research has been done on myeloid neoplasms related to pediatric DS; we now emphasize the importance of reporting similar findings in adult patients, so as to be able to better delineate the course and subsequent management of this under-recognized condition in later life.
